# SRC plays a specific role in the cross-talk between apoptosis and autophagy via phosphorylation of a novel regulatory site on AMPK

**DOI:** 10.1080/27694127.2022.2047266

**Published:** 2022-03-22

**Authors:** Ming Zhao, Darren Finlay, Robert Liddington, Kristiina Vuori

**Affiliations:** Cancer Center, Sanford Burnham Prebys Medical Discovery Institute, 10901 N. Torrey Pines Road, La Jolla, CA 92037, USA

**Keywords:** AMPK, apoptosis, autophagy, cell attachment, cell detachment, phosphorylation, PTK2/FAK, SRC

## Abstract

Cell detachment from the extracellular matrix (ECM) typically promotes cell death via a form of apoptosis known as anoikis. However, in tumor cells, detachment can also induce cell survival, utilizing a process known as macroautophagy/autophagy, which involves degradation and removal of apoptotic proteins as well as rewiring of metabolic pathways so that cells can survive under stress. The crosstalk between the competing processes of anoikis and autophagy is only partially understood but may be critical for the design of multi-drug therapeutic strategies. Here, we summarize our recent studies, which reveal a direct regulatory link between a major mediator of cell survival in adherent cells, the ECM-integrin-activated dual tyrosine kinase complex of SRC and PTK2/FAK, and a major regulator of cell metabolism and autophagy, AMP-activated protein kinase (AMPK). We identify a novel SRC phosphorylation site on AMPK and demonstrate that this phosphorylation event plays key roles in AMPK regulation, autophagy induction, and cell survival.

It has been shown previously that cell detachment-induced autophagy offers a survival mechanism during cancer cell dissemination to distant sites. Subsequently, autophagy presumably needs to be deactivated to maintain cellular homeostasis when cancer cells reach their metastatic sites to colonize and grow. But how autophagy is fine-tuned during this process of cell detachment-reattachment is not clear. Our recent study [1] has yielded new insights into the understanding of the role that the SRC-PTK2 complex plays in regulating AMPK and autophagy during cell detachment and reattachment (Fig. 1). We first showed that while cell detachment from the ECM induces activation of both AMPK and autophagy, the latter is significantly impaired in detached cells lacking AMPK. Moreover, activation of both AMPK and autophagy is enhanced and sustained when either SRC kinase activity is inhibited chemically, or when either SRC kinases or PTK2 are genetically deleted. Also, in cells lacking either SRC kinases or PTK2, chemical AMPK activators are able to induce higher phosphorylation levels at PRKAA-T172 (the site for activation in the alpha-subunit of AMPK), as well as on ULK1 (a downstream substrate of active AMPK), further indicating an inhibitory effect of the SRC-PTK2 complex on AMPK signaling. We also examined the role of autophagy in cell survival. We observed that detached cells wherein autophagy has been inhibited undergo anoikis at a greater rate than those with autophagy unperturbed. This supports the notion that induction of autophagy may be a necessary mechanism to protect detached cells from cell death and possibly be hijacked in metastatic cancer cells.

**Figure 1.**
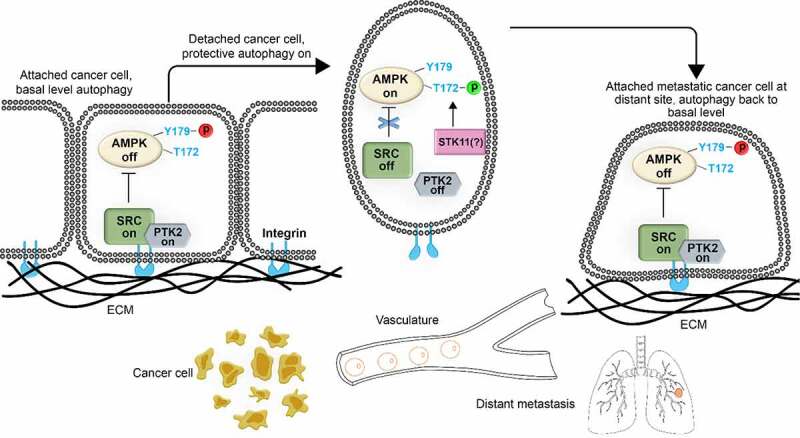
A proposed model for AMPK-autophagy regulation by SRC during cell detachment and reattachment. When cells are detached, SRC is inactive, and AMPK becomes activated to induce autophagy that offers protection against apoptosis (anoikis). When cells are re-attached, SRC is activated, suppresses AMPK activity, and brings autophagy down to a basal level, allowing cells to proliferate.

We next determined that SRC directly phosphorylates AMPK *in vitro*. By various biochemical, genetic, and proteomic methods, we demonstrated that SRC phosphorylates the catalytic alpha-subunit of AMPK, PRKAA, at a novel site, Y179, which is exposed in the inactive, open, state of the AMPK catalytic domain, but buried in the protein interior in the active, closed state. Y179 is adjacent to but distinct from the T172 activation loop of the kinase domain. Our modeling studies suggest that phosphorylation of Y179 destabilizes the closed, active conformation of the kinase domain, thereby promoting an open, inactive AMPK, irrespective of whether T172 is phosphorylated. We tested this hypothesis by reconstituting *PRKAA* knockout cells with a phospho-mimetic PRKAA mutant, Y179E, which results in defective AMPK activation as measured by diminished phosphorylation at PRKAA-T172 and its downstream substrate, ULK1. (It is also conceivable that ADP-SRC binds irreversibly to the phosphomimetic residue, because it lies within an authentic recognition motif, and blocks AMPK activation sterically. Indeed, we note that SRC co-immunoprecipitates more readily with the Y179E mutant compared with the wild-type protein).

Similar experiments using a non-phosphorylatable Y179F mutant show a higher level of T172 phosphorylation than for the wild-type protein. Notably, however, this mutant is less catalytically active toward its downstream substrates. Structural modeling suggests that the mutation makes the T172 phosphorylation site more accessible to its cognate kinase, either by having a more flexible activation loop or by promoting the open conformation of the bilobal domain; but that its reduced activity toward substrate arises from a destabilization of the interaction of the adjacent K141 with ATP in the AMPK-substrate complex.

We also examined SRC regulation of autophagy in cancer cell survival and drug resistance. To this end, we found that while SRC inhibitors promote cell death in cancer cells harboring oncogenic SRC, simultaneous inhibition of autophagy (by hydroxychloroquine or by *ATG5* or *ATG7* knockout) promotes apoptosis of cancer cells even further. This suggests that induction of autophagy upon SRC inhibitor treatment may contribute to drug resistance.

Taken together, our studies add to the growing literature demonstrating the importance of autophagy in promoting cell survival in detached, potentially metastatic, cancer cells, and further suggest that autophagy is involved in preventing cytotoxicity induced by clinically relevant agents. While our studies add an important mechanistic insight into the role of the SRC-PTK2 signaling complex in autophagy regulation *vis à vis* AMPK, a comprehensive understanding of the molecular and cellular mechanisms of autophagy regulation, and the significance of autophagy in cancer progression and drug resistance merits further research.
